# Sex- and age-specific effects of energy intake and physical activity on sarcopenia

**DOI:** 10.1038/s41598-020-66249-6

**Published:** 2020-06-17

**Authors:** Yu Jin Cho, Youn-Hee Lim, Jae Moon Yun, Hyung-Jin Yoon, Minseon Park

**Affiliations:** 1 Department of Family Medicine, Seoul National University, College of Medicine, Seoul, Korea; 2 Institute of Environmental Medicine, Seoul National University Medical Research Center, Seoul, Korea; 3 Department of Biomedical Engineering, Seoul National University College of Medicine, Seoul, Korea; 4 Bio-MAX Institute, Seoul National University, Seoul, Korea

**Keywords:** Lifestyle modification, Nutritional supplements

## Abstract

Sarcopenia is a common health issue that is not limited to only elderly patients. However, many studies have reported factors to prevent sarcopenia only in susceptible groups. This study evaluates the relationship of the total energy intake to basal metabolic rate ratio (EI/BMR) and physical activity (PA) with sarcopenia. A second aim was to analyze the interaction between EI/BMR and PA by sex and age. We analyzed 16,313 subjects aged ≥ 19 years who had dual‒energy X-ray absorptiometry data. Sarcopenia was defined as appendicular lean mass/weight (%) that was 1 standard deviation below the sex-specific mean value for a young reference group. Multivariate logistic regression analysis was used to examine the interaction between EI/BMR and PA. In this study, as EI/BMR increased, the risk of sarcopenia decreased, particularly in the older groups. Both high PA and high EI/BMR were independently related to the reduced risk of sarcopenia and showed additive effects on reducing the risk in young male and older groups. However, high PA was associated with an increased risk of sarcopenia in the young female group with low energy intake. Our findings suggest that an adequate balance between energy intake and PA is related to a low risk of sarcopenia, especially in young females.

## Introduction

Sarcopenia, an age-related decline in skeletal muscle mass and strength^1^, is an important health issue that has recently attracted increasing attention. In 2000, the medical cost of sarcopenia increased to close to 1.5% of the total national healthcare expenditure in the USA^2^, and in 2017, the World Health Organization listed sarcopenia as a disease and designed an International Classification of Disease, Tenth Revision, Clinical Modification code for it (ICD-10-CM, M62.84)^3^.

Although sarcopenia was first described as a risk factor for physical frailty and disabilities, it is now studied not only in geriatric medicine, but also in a wide range of specialties^4–7^. Many studies have reported that sarcopenia is an important factor in the development of various diseases such as metabolic syndrome, cardiovascular diseases (CVD), and osteoporosis^8–12^.

Sarcopenia is known to affect the morbidity and mortality of vulnerable groups such as cancer survivors, those with chronic diseases, and older people aged ≥ 65. Many reports have mainly referred to various risk factors of sarcopenia in these susceptible groups. However, sarcopenia currently affects all ages. Various risk factors including personal factors, chronic health conditions, and lifestyle behaviors are known factors influencing sarcopenia^13^. Moreover, sarcopenia is related to disease susceptibility even in healthy young adults and the elderly^14^. While sarcopenia in older people is primarily associated with frailty, sarcopenia in younger people is associated with metabolic syndrome^15^. In 2019, Goh *et al*. reported that sarcopenia was significantly associated with the severe nonalcoholic fatty liver disease (NAFLD) independent of visceral fat and other metabolic confounders, and the association was significantly stronger in younger age group^16^.

In Korea, there has been a rapid change in lifestyle behaviors since 1980 following the introduction of a westernized diet and the increased popularity of motor vehicles. Changes in dietary habits and decreased PA might have resulted in sarcopenia^17^. According to the National Institutes of Disease Control, the annual meat consumption per person increased from 11.3 kg in the 1980s to 46.8 kg in 2015, and the rice consumption decreased from 132.4 kg to 62.9 kg. In addition, the obesity rate increased from 28.1% in 1998 to 37.3% in 2017, especially in young adults. Moreover, the ‘walking practice rate’, which represents the level of PA, decreased by 20% from 60.7% in 2005 to 40.2% in 2018. (Korea Centers for Disease Control and Prevention 2017).

In Korea, a previous study reported that the prevalence of sarcopenia was 19.2%, 29.1%, and 42.3% among individuals in the 20–39, 40–64, and over 65-year age groups, respectively^18^. Therefore, a study of sarcopenia including young adults, is needed.

The basal metabolic rate (BMR), which is influenced by  age, sex, height and weight, is defined as the daily rate of energy expenditure needed to preserve vital functions at rest^19^, and reflects whole-body energy metabolism^20^. The BMR represents 35%‒70% of the total energy requirement of an individual, and in sedentary individuals, the BMR accounts for 60%‒70% of the total energy expenditure^21^. The BMR is relatively constant among population groups of a given age and sex, so it could be used to determine the energy requirements of each person, as it reflects body weight and PA level^22^.

Therefore, this study mainly aimed to evaluate the correlation between sarcopenia and the ratio of total energy intake to BMR (EI/BMR) and PA and then to analyze how the interaction between these two factors differs among different sex and age groups in the Korean population.

## Results

### Basal characteristics of the study participants

This study included a total of 16,313 participants aged ≥ 19 years, with 8,092 participants aged <50 years (young group) and 8,221 participants aged >50 years (older group). The prevalence of sarcopenia was 9% (732) in the young group and 21.5% (1,767) in the older group.

Table 1 shows that the mean age of the participants was approximately 2–3 years higher in the sarcopenia group than in the non-sarcopenia group. Alcohol consumption was not different between the young sarcopenia group and young non-sarcopenia group, while the older non-sarcopenia group consumed more alcohol than the older sarcopenia group. With respect to smoking, the young sarcopenia group smoked more than the young non-sarcopenia group; however, the older non-sarcopenia group smoked more than the older sarcopenia group. The number of participants with abdominal obesity was higher in the sarcopenia group than in the non-sarcopenia group and higher in the older group than in the young group, regardless of sex. In addition, metabolic diseases, such as HTN, DM, or CVD, were more prevalent in the older sarcopenia group than in the older nonsarcopenia group, whereas only HTN was more prevalent in the young sarcopenia group than in the young nonsarcopenia group.

**Table 1:** Basal characteristics of stratified study participants (≥19 years).

Characteristics	Age <50 (N = 8,092)						Age ≥ 50 (N = 8,221)					
	Male (N = 3,127)		*P-value*	Female (N = 4,965)		*P-value*	Male (N = 3,449)		*P-value*	Female (N = 4,772)		*P-value*
	Sarcopenia (N = 307)	Normal (N = 2,820)		Sarcopenia (N = 425)	Normal (N = 4,540)		Sarcopenia (N = 695)	Normal (N = 2,754)		Sarcopenia (N = 1,072)	Normal (N = 3,700)	
Age (continuous)	37.5 ± 7.6	35.8 ± 8.2	0.001	37.8 ± 8.1	36.0 ± 8.2	<0.0001	67.0 ± 9.0	63.5 ± 8.7	<0.0001	65.2 ± 9.0	63.5 ± 9.4	<0.0001
Smoking status			0.005			<0.0001			<0.0001			0.054
Non-smoker	48 (15.7)	623 (22.2)		342 (80.7)	3,917 (86.6)		118 (17.2)	461 (16.8)		993 (93.5)	3,358 (91.5)	
Past smoker	48 (15.7)	310 (11.0)		31 (7.3)	143 (3.2)		213 (31.0)	644 (23.5)		17 (1.6)	57 (1.6)	
Current smoker	209 (68.5)	1,877 (66.8)		51 (12.0)	465 (10.3)		356 (51.8)	1,636 (59.7)		52 (4.9)	256 (7.0)	
Alcohol consumption			0.172			0.529			<0.0001			0.017
No	34 (11.2)	247 (8.8)		100 (23.7)	1,009 (22.4)		219 (32.0)	657 (24.0)		607 (57.5)	1,956 (53.4)	
Yes	270 (88.8)	2,554 (91.2)		322 (76.3)	3,504 (77.6)		465 (68.0)	2,080 (76.0)		448 (42.5)	1,707 (46.6)	
Hypertension	44 (14.3)	149 (5.2)	<0.0001	17 (4.0)	124 (2.7)	<0.0001	389 (56.0)	904 (32.8)	<0.0001	574 (53.5)	1,353 (36.6)	<0.0001
Diabetes	11 (3.6)	55 (1.9)	0.059	8 (1.9)	56 (1.2)	0.257	170 (24.5)	384 (13.9)	<0.0001	180 (16.8)	424 (11.5)	<0.0001
CCVD	2 (0.7)	19 (0.7)	0.964	1 (0.2)	20 (0.4)	0.533	124 (17.8)	210 (7.6)	<0.0001	103 (9.6)	217 (5.9)	<0.0001
Cancer	1 (0.3)	4 (0.1)	0.444	9 (2.1)	38 (0.8)	0.009	19 (2.7)	89 (3.2)	0.501	36 (3.4)	138 (3.7)	0.568
BMI (kg/m2)	27.4 ± 3.7	23.8 ± 3.0	<0.0001	26.2 ± 4.4	22.2 ± 3.2	<0.0001	25.5 ± 2.9	23.2 ± 2.8	<0.0001	26.5 ± 3.2	23.53 ± 2.9	<0.0001
Abdominal obesity			<0.0001			<0.0001			<0.0001			<0.0001
Yes	174 (56.8)	523 (18.6)		176 (41.4)	511 (11.3)		411 (59.2)	611 (22.3)		678 (63.5)	1,138 (30.9)	
No	132 (43.1)	2,289 (81.4)		249 (58.6)	4,010 (88.7)		283 (40.8)	2,135 (77.8)		390 (36.5)	2,550 (69.1)	
Total energy intake (kcal)	2314 ±856	2438 ±844	0.015	1641 ±616	1741 ±645	0.002	1900 ±667	2110 ±729	<0.0001	1489 ±526	1578 ±565	<0.0001
Protein (g)	85.6 ± 40.6	89.9 ± 41.8	0.081	60.43 ± 27.89	63.47 ± 29.31	0.039	66.1 ± 32.5	73.1 ± 34.7	<0.0001	50.6 ± 26.7	52.2 ± 25.6	0.072
Carbohydrate (g)	336.5 123.1	361.3 ±124.0	0.0009	263.6 ±96.7	283.8 ±108.1	0.0002	321.1 ±105.6	351.5 ±115.6		274.6 ±97.3	295.2 ±108.0	<0.0001
BMR (kcal)	1681 ±165	1612 ±136	<0.0001	1278 ±149	1224 ±110	<0.0001	1405 ±145	1372 ±141	<0.0001	1074 ±130	1036 ±134	<0.0001
Intake/BMR ratio	1.38 ± 0.52	1.51 ± 0.53	<0.0001	1.29 ± 0.50	1.43 ± 0.54	<0.0001	1.35 ± 0.44	1.53 ± 0.51	<0.0001	1.39 ± 0.48	1.53 ± 0.54	<0.0001
Strength exercise			<0.0001			0.902			0.020			0.010
Yes	95 (31.2)	1,204 (42.8)		78 (18.4)	844 (18.6)		200 (29.0)	924 (33.7)		115 (10.8)	507 (13.8)	
No	210 (68.9)	1,608 (57.2)		346 (81.6)	3,684 (81.4)		489 (71.0)	1,819 (66.3)		951 (89.2)	3,168 (86.2)	
Flexibility exercise			0.008			0.232			0.137			0.050
Yes	153 (50.2)	1,634 (58.1)		223 (52.6)	2,518 (55.6)		316 (45.9)	1,345 (49.0)		419 (39.3)	1,568 (42.7)	
No	152 (49.8)	1,178 (41.9)		201 (47.4)	2,010 (44.4)		373 (54.1)	1,398 (51.0)		647 (60.7)	2,107 (57.3)	
MET-h/week	42.9 ± 75.2	59.7 ± 87.9	0.001	40.7 ± 58.7	42.3 ± 69.1	0.635	43.9 ± 64.4	66.1 ± 90.6	<0.0001	41.0 ± 75.2	51.9 ± 85.4	0.0002

Std. Dev.

CCVD (cardio-cerebrovascular disease): stroke, MI, angina; cancer: stomach, liver, colorectal, breast, cervical, lung cancer.

The EI/BMR of the non-sarcopenia group was higher than that of the sarcopenia group in both age groups regardless of sex (p < 0.001). In addition, the non-sarcopenia group exercised more than the sarcopenia group, except for the young female group. When we expressed PA in MET‒h, the sarcopenia group engaged in less PA than the non-sarcopenia group. When we divided each group into male and female groups, this difference was the greatest in the older male group (43.94 ± 64.38 vs 66.06 ± 90.61 MET‒h/week, p < 0.001). However, in the young female group, PA was not different between the sarcopenia and non-sarcopenia groups (40.65 ± 58 vs 42.30 ± 69.10 MET‒h/week, p = 0.635).

### Predicted risk probability of sarcopenia according to EI/BMR and physical activity

**Figure 1:**
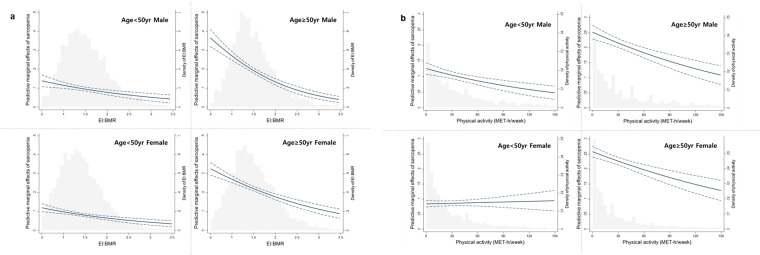
Predictive plots of the risk of sarcopenia with histogram summarizing the distribution of the a) EI:BMR, b)METs.

Figures 1a shows the predicted risk probability of sarcopenia according to EI/BMR. As EI/BMR increased, the predicted risk probability of sarcopenia decreased, and it appeared more prominent in the older groups.

In the young male and female group, the predicted risk probability of sarcopenia decreased from 0.11 to 0.05 and 0.1 to 0.04 when the energy intake increased from 1 to 3 times of the BMR; in contrast, in the older male and female groups, the predicted risk probability decreased from 0.28 to 0.05 and 0.2 to 0.1 when the energy intake increased from 1 to 3 times of the BMR, respectively.

As shown in Fig. 1b, the predicted risk probability of sarcopenia also decreased with increasing PA level. Interestingly, in the young female group, the predicted risk probability of sarcopenia remained almost unchanged.

### Multivariate logistic regression analysis of sarcopenia according to each influencing factor

The associations between influencing factors and the risk of sarcopenia with or without adjustment for confounding factors are presented in Table 2.

**Table 2:** Adjusted ORs for sarcopenia according to age group.

		Univariate				Multivariate			
		Age <50		Age ≥ 50		Age <50		Age ≥ 50	
		OR	*P-value*	OR	*P-value*	OR	*P-value*	OR	*P-value*
Age	M	1.025	0.001	1.045	<0.0001	1.013	0.096	1.026	<0.0001
	F	1.029	<0.0001	1.019	<0.0001	1.029	<0.0001	1.006	0.132
Abdominal obesity	M	5.769	<0.0001	5.074	<0.0001	5.460	<0.0001	5.111	<0.0001
	F	5.546	<0.0001	3.893	<0.0001	5.460	<0.0001	3.586	<0.0001
Protein (1 g/kg/day)	M	0.530	<0.0001	0.419	<0.0001	0.396	<0.0001	0.434	<0.0001
	F	0.476	<0.0001	0.548	<0.0001	0.325	<0.0001	0.523	<0.0001
Carbohydrate (100 g)	M	0.843	0.001	0.774	<0.0001	0.818	0.008	0.867	0.032
	F	0.826	<0.0001	0.822	<0.0001	0.744	0.004	0.707	<0.0001
Intake:BMR ratio (0.5)	M	0.780	<0.0001	0.656	<0.0001	0.654†	0.001	0.477†	<0.0001
	F	0.776	<0.0001	0.765	<0.0001	0.508†	<0.0001	0.367†	<0.0001
Strength exercise	M	0.604	<0.0001	0.805	0.020	0.651	0.001	0.928	0.455
	F	0.984	0.902	0.755	0.011	0.958	0.751	0.852	0.159
Flexibility exercise	M	0.725	0.008	0.880	0.137	0.740	0.015	0.936	0.475
	F	0.885	0.233	0.869	0.050	0.860	0.143	0.951	0.500
MET-h/week (14)	M	0.954	0.001	0.944	<0.0001	0.956	0.002	0.958	<0.0001
	F	0.995	0.645	0.974	<0.0001	0.990	0.372	0.982	0.010

Univariate: no adjustment.

Multivariate: adjusted for metabolic disease (HTN, DM, CCVD), lifestyle factor (smoking, drinking), total energy intake, physical activity (MET-h/week), age.

^†^adjusted for metabolic disease (HTN, DM, CCVD, age), lifestyle factor (smoking, drinking), carbohydrate intake, protein intake, age.

CCVD, cardio-cerebrovascular disease; DM, diabetes mellitus; HTN, hypertension; OR, odds ratio.

As age increased, the risk of sarcopenia increased. In the young group, those taking 50% more energy than the BMR showed an inverse correlation between the risk of sarcopenia (OR 0.780 p < 0.0001, OR 0.776 p < 0.0001, male and female groups, respectively). In the older group, similar results were also observed (OR 0.656 p < 0.0001 and OR 0.765 p < 0.0001, in the male and female groups, respectively). Consuming optimal dietary carbohydrates as well as taking in the optimal amount of dietary protein was also inversely associated with the risk of sarcopenia in all groups (Table 2). A PA level as high as 14 MET-h/week also had a significant inverse association with the risk of sarcopenia, but in the young female group, such high levels of PA did not affect the risk of sarcopenia (OR 0.995, p = 0.645).

To determine the independent association between sarcopenia and EI/BMR and PA, multivariate analyses were performed. The significant effect of EI/BMR on the risk of sarcopenia was supported, and the effect was highest in the older female group (OR 0.367 p < 0.0001). The effect of protein intake and carbohydrate intake on the risk of sarcopenia also remained significant, and the effect of protein was greater, especially in the young group (OR 0.396, p < 0.0001 and OR 0.325, p < 0.0001, in the male and female groups, respectively). After adjustment for all confounding factors, the associations between the influential factors and the risk of sarcopenia were similar to the results of the univariate model (Table 2).

### Adjusted OR of sarcopenia according to the interaction between EI/BMR and physical activity

**Figure 2:**
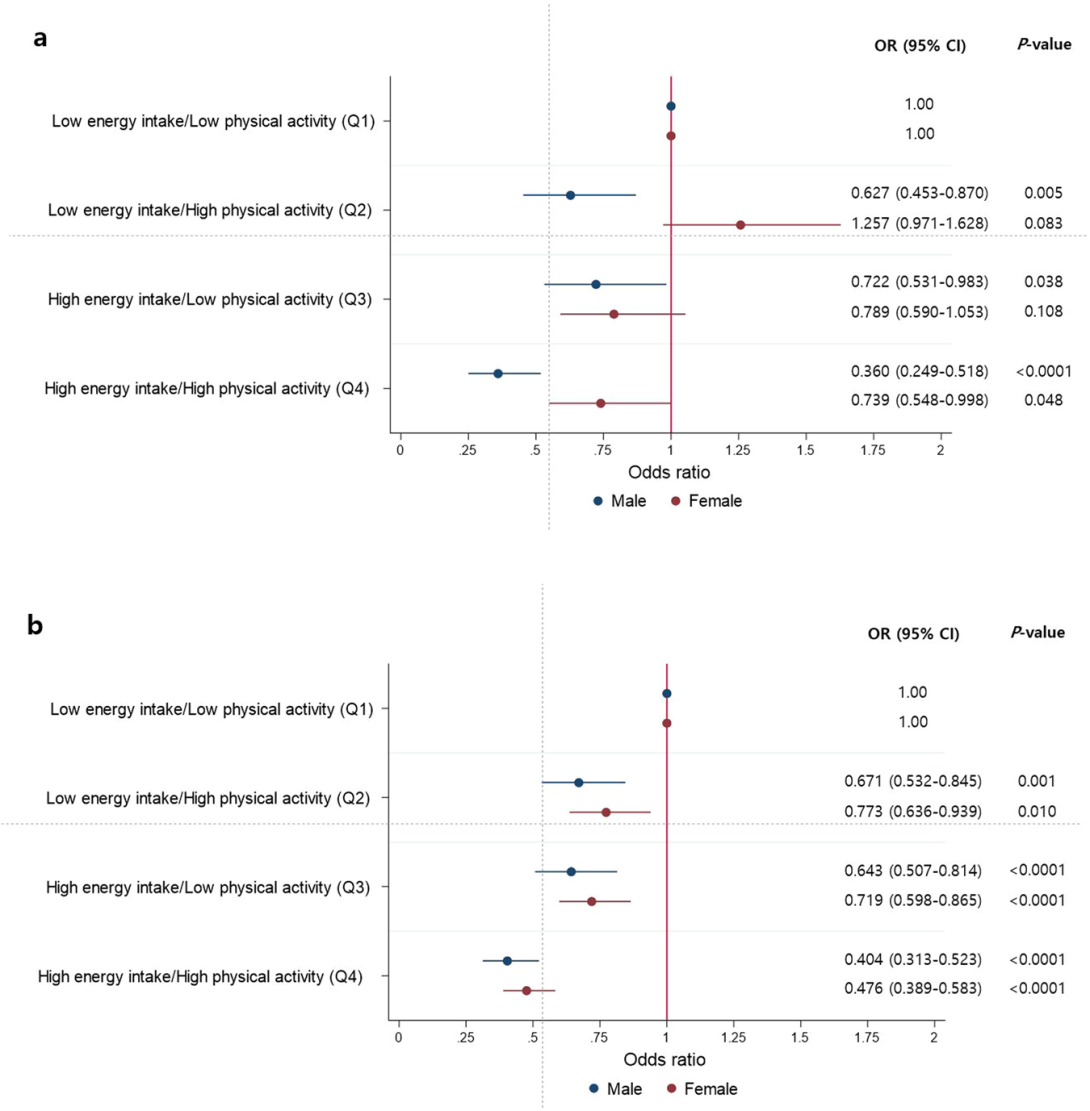
Relative risk of sarcopenia according to energy intake and physical activity level (**a**) age <50, (**b**) age ≥ 50.

To analyze the age- and sex-specific differential effects of EI/BMR and PA on the risk of sarcopenia, we used post-estimation model of logistic regression. After adjusting for age, lifestyle factors (smoking, alcohol consumption) and chronic diseases (HTN, DM, CCVD), the results in both sexes in the same age groups are shown in one graph using a coefficient plot (Fig. 2).

After setting the median values of the EI/BMR (1.43 and 1.39 in male and female groups, respectively) and PA (29.7 and 19.8 in male and female groups, respectively, MET-h/week), we defined “low” as lower than the median and “high” as higher than the median value.

**Figure 3:**
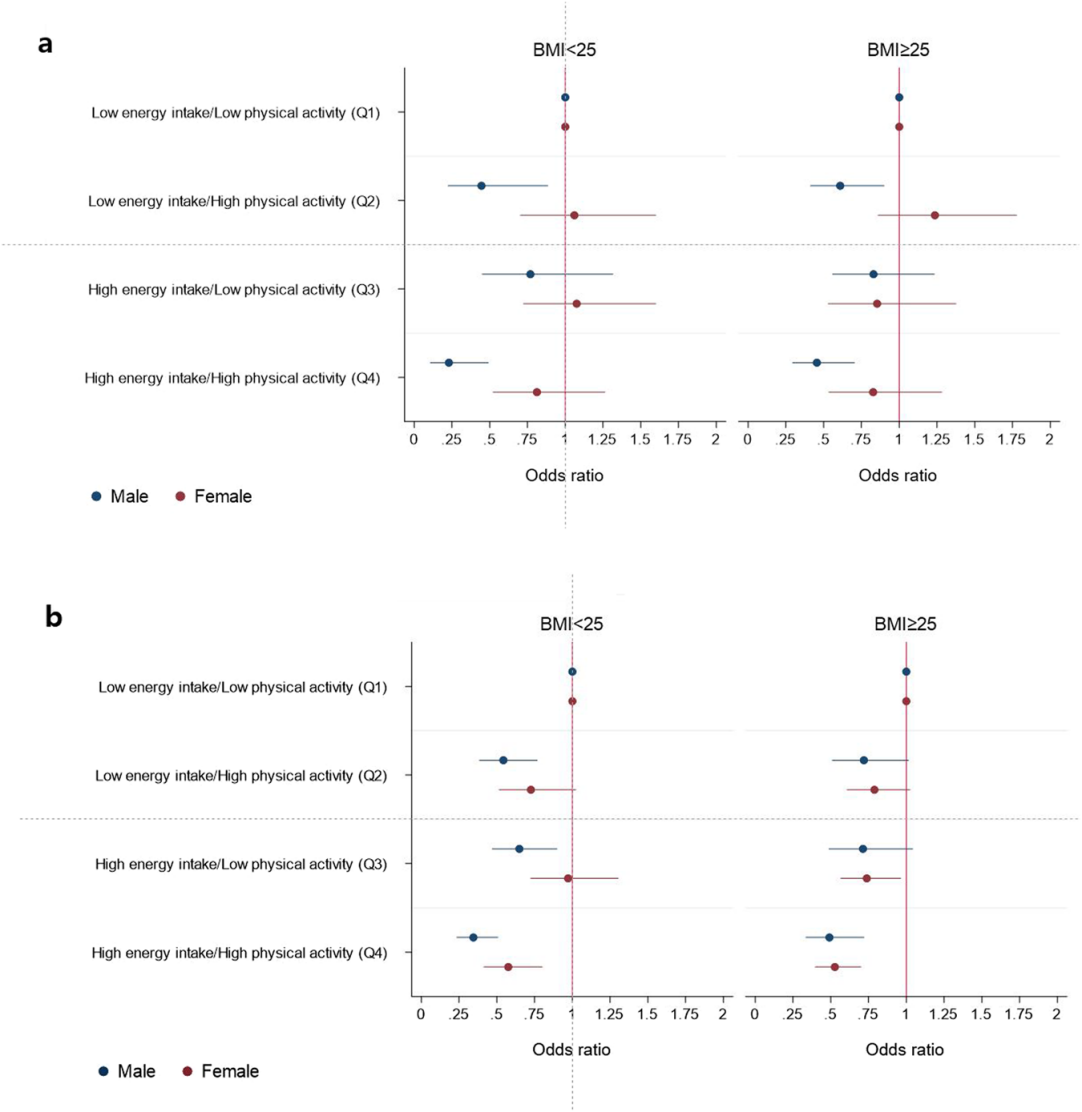
Relative risk of sarcopenia according to BMI (**a**) age <50, (**b**) age ≥ 50.

If the risk of participants with “low” energy intake and “low” levels of PA was set at 1, making this the reference group (Q1), the relative OR of the “low” energy intake and “high” PA group (Q2), “high” energy intake and “low” PA group (Q3), and “high” energy and “high” PA group (Q4) are shown in Fig. 3.

In the young male group, we compared Q2 with Q3. Figure 2 shows that a high level of PA is related to a lower OR of sarcopenia than is high energy intake (OR 0.627, 95% CI 0.453–0.870; OR 0722, 95% CI 0.531–0.983, in groups Q2 and Q3, respectively), and the additional effect of a high level of PA on high energy intake on the reduction in the OR of sarcopenia (Q4 vs Q3) was similar to that of low energy intake (Q2 vs Q1) (OR 0.722, 95% CI 0.531–0.983; OR 0.360, 95% CI 0.249–0.518, in groups Q3 and Q4; OR 0.627, 95% CI 0.453–0.870; OR 1 (reference value) in groups Q2 and Q1, respectively). However, in the young female group, the combined effect of low energy intake and a high level of PA showed an increased in the OR of sarcopenia by 0.257 compared to the reference group (OR 1.257, 95% CI 0.971–1.628, in Q2) (Fig. 2a).

In the older group, the effect of EI/BMR and PA on the OR of sarcopenia were different from those in the young group. High PA and high EI/BMR were independently related to the reduced risk of sarcopenia and showed additive effects on the reduction in the risk of sarcopenia (Q4 OR 0.404, 95% CI 0.313–0.523, male group; Q4 OR 0.476, 95% CI 0.389–0.583, female group, respectively).

### Different effects of the interaction between EI/BMR and physical activity on the risk of sarcopenia according to obesity status

To analyze the difference in the interaction between EI/BMR and PA according to obesity status, we divided the entire population into lean (BMI < 25 kg/m^2^) and overweight (BMI ≥ 25 kg/m^2^) groups. Each group was adjusted by age, lifestyle factors (smoking, alcohol consumption) and chronic diseases (HTN, DM, CCVD), and all group in the same age groups are shown on one graph using a coefficient plot (Fig. 3).

In the young group, the effects of EI/BMR and PA on the risk of sarcopenia were similar regardless of obesity status. The combination of low energy intake and a high level of PA non-significantly increased the OR of sarcopenia in lean and in overweight young female groups. Additionally, in the lean young female group, high energy intake with a low level of PA, also led to a nonsignificantly increased OR of sarcopenia compared to the PA reference group (Fig. 3a).

**Figure 4:**
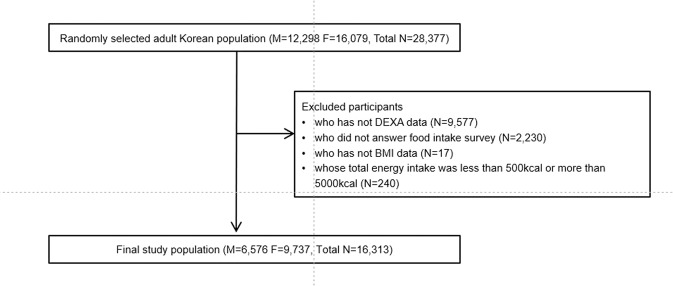
Study population. KNHANES, Korean National Health and Nutrition Examination Survey.

In the older group, the effect of EI/BMR and PA on the OR of sarcopenia were similar to those in total older group, regardless of obesity status (Fig. 4b).

## Discussion

In this study, we found that both a high level of PA (≥19.8 MET-h/week, median value) and high EI/BMR (≥1.39 EI/BMR, median value) showed significant positive association with the risk of sarcopenia in the young male group and older group. However, a high level of PA with low energy intake was associated with a high risk of sarcopenia in the young female group.

Because sarcopenia is an age-related process, with a marked loss of muscle mass and strength occurring around age 50^23^, we analyzed the effect of energy intake and PA on sarcopenia and stratified the population based on age, with the cutoff value 50 years. In this study, the prevalence of sarcopenia was approximately 9% in the young group and approximately 21% in the older group as in a previous study^24^.

Previous studies demonstrated that adequate energy intake has been negatively associated with the sarcopenic index^25–27^, and most studies seemed to be in agreement that the higher the energy intake, the better the effect on the risk of sarcopenia^26,28,29^. Some studies reported that PA, especially moderate-to-vigorous intensity, was associated with a reduced risk of sarcopenia^30–34^. In contrast, another study suggested no association between PA and sarcopenia^35^.

In this cross‒sectional observation study, the predicted risk probability of sarcopenia was affected by EI/BMR and PA in all groups regardless of sex and age. However, the effect size was different according to age group. The maximum predicted risk probability of sarcopenia in the older male group was twice as high as that in the young male group, but the minimum predicted risk probability of sarcopenia according to the increase in EI/BMR was similar in the two groups. However, in the female group, the minimum predicted risk probability of sarcopenia according to the increase in EI/BMR was higher in the older group. In our study population, the average energy intake of older men and women was similar, but the proportion of individuals who engaged in PA and the duration of PA were reduced in the older female group compared with the older male group (Supplementary Table S1). This means that if enough energy is consumed, the minimum predicted risk probability of sarcopenia in the older group can be reduced compared to that in the young group. However, excess energy intake could also turn into body fat, which might result in a higher minimum risk probability of sarcopenia in older women.

Similarly, PA also showed an inverse correlation with the predicted risk probability of sarcopenia, except in the young female group. This result was consistent with those of previous studies showing that PA had a positive effect on muscle mass and function in people aged ≥ 60 years^36,37^. However, the predicted risk probability of sarcopenia did not decrease in the young female group with increasing PA, and even a high level of PA, when combined with low energy intake, was related to a non-significantly increased risk of sarcopenia in the young female group.

To our knowledge, this is the first study showing unfavorable results with regard to sarcopenia in the young female group who engaged in high levels of PA and had low energy intake. Therefore, an explanation for this association is somewhat elusive. First, the nature of our study population influences this finding. In our study, in all groups except the young female group, EI/BMR increased as the level of PA increased (Supplementary Fig. S1). Second, many studies have reported that oxidative stress can cause a loss of muscle mass and strength^38^. Early studies demonstrated that high intensity exercise increases oxidative stress in muscle cells^39,40^. As PA increases, oxidative stress and inflammation increase^41,42^, and young women with low energy intake are more likely to experience undernutrition^43^. To eliminate these reactive oxygen species and inflammation, the immune system needs a large quantity of energy‐rich fuel^44^, mainly glucose. The lack of energy intake, especially carbohydrates, results in the degradation of muscles due to the elimination of inflammation through gluconeogenesis^44,45^. Therefore, an imbalance in energy intake and expenditure might be a potential explanation for the higher risk of sarcopenia in the more active young female group.

Many studies have reported the importance of adequate protein intake for the prevention and management of sarcopenia^46–48^, but few studies have reported the effects of carbohydrate and total energy intake on the risk of sarcopenia^49^. In this study, protein intake was associated with a lower risk of sarcopenia after adjusting for influential factors, which is in line with previous results^47,50^. Adequate carbohydrate intake and protein intake also showed favorable effects on the risk of sarcopenia.

We tried to analyze the interactive effect of EI/BMR and PA on the risk of sarcopenia stratified by age and sex. Both a high level of PA and high EI/BMR were positively associated with the risk of sarcopenia in men, regardless of age group. However, contrary to the results of previous studies, a high level of PA with low energy intake was associated with an elevated risk of sarcopenia in the young female group. In the young female group, EI/BMR significantly influenced the effect of PA on the risk of sarcopenia, while in other groups, an additive effect of EI/BMR and PA on the risk of sarcopenia was observed. The different effects of sex hormones on muscle mass and function^13,51–54^ and a greater discrepancy between EI/BMR and PA (including strength and flexibility exercises) in the young female group might be a possible explanation for these associations (Supplementary Fig. S1). Further prospective studies are needed to clarify these findings.

In a previous study, the combined effect of energy intake and PA on sarcopenia was potentially different between the overweight and lean groups^27^. Therefore, we analyzed the effect of energy intake and PA on sarcopenia stratified by BMI with a cutoff value of 25 kg/m^2^, and the combined effects of EI/BMR and PA were not different between the overweight and lean groups, regardless of sex and age group (Supplementary Fig. S2).

This study has some potential limitations. First, the cross-sectional nature of this study makes identifying a cause-and-effect relationship difficult. Second, the use of self-administered questionnaires to obtain detailed information on lifestyle behaviors and disease history might limit the accuracy of our results. In the same manner, the 24-h recall method of assessing dietary variables may not be representative of the exact habitual diet.

Despite these limitations, the present study was a large, population-based study analyzing the associations of EI/BMR and PA with the risk of sarcopenia. The study also investigated the effect of the interaction between EI/BMR and PA on sarcopenia stratified by sex and age group. We showed that sufficient energy intake is more strongly associated with a lower risk of sarcopenia than is a high level of PA, which is usually recommended for the prevention of chronic noncommunicable diseases in women and older people^55,56^. Moreover, a high level of PA was not related to a reduced risk of sarcopenia in the young female group.

In conclusion, our study suggested that an adequate balance between energy intake and PA is related to a lower risk of sarcopenia, especially in the young female group. However, in the young male group, engaging in a high level of PA is a more important means of decreasing the risk of sarcopenia, and in the older group, consuming sufficient energy or engaging in a high level of PA is enough to prevent sarcopenia. Further prospective studies are needed to validate an age group‒specific strategy to prevent sarcopenia.

## Materials and Methods

This study was a secondary analysis of data obtained from the 2008 to 2011 Korean National Health and Nutrition Examination Survey (KNHANES IV and V), a nationally representative survey conducted by the Korean Ministry of Health and Welfare. Written informed consent was given by all participants, and the protocol for KNHANES IV and V was approved by the Institutional Review Board of the Korean Centers for Disease Control and Prevention.

A whole-body, dual‒energy X-ray absorptiometry (DXA) scan was performed on individuals ≥ 10 years old between July 2008 and May 2011.

### Participants

We selected participants aged 19 years or older who had DXA and body mass index (BMI) data. We excluded participants who did not answer the food intake survey and participants whose total energy intake was <500 kcal or >5000 kcal a day. Finally, 16,313 participants were included in our analysis (Fig. 4).

### Measurements

#### Sarcopenia

In the KNHANES, whole-body DXA examinations were conducted with a QDR4500A apparatus (Hologic Inc., Bedford, MA, USA). The data included values for bone mineral content (g), bone mineral density (g/cm2), fat mass (g), lean mass (including bone mineral content [g]), and fat percentage of whole body and anatomical regions.

Sarcopenia was defined as an appendicular skeletal muscle mass (ASM)/weight (Wt) (%) that was 1 standard deviation or more below the sex-specific mean value for a younger reference group (20 s and 30 s) which enables the detection of risk factors for metabolic diseases^18,57,58^ (<0.305 and <0.239, men and women, respectively)

#### Influencing factors

Behavioral factors, including smoking or drinking, were assessed by self-administered questionnaires. The participants who had been diagnosed with hypertension (HTN), diabetes mellitus (DM), CVD, or cancer were defined as those who answered “yes” to the question, “Have you ever been diagnosed with ‘disease’ by a doctor?” Cardiocerebrovascular disease (CCVD) included stroke, myocardial infarction (MI), and angina, and cancer included stomach, liver, colorectal, breast, cervical, and lung cancer.

Abdominal obesity as determined by waist circumference was defined as ≥ 90 cm for men and ≥ 85 cm for women^59^. The BMR was calculated using height, weight, and age by the Harris–Benedict equation. Self-administered questionnaires for energy intake and PA were used. Energy intake was measured by the single 24-h dietary recall method. Trained interviewers investigated all food consumed by the study subjects in the last 24 hours using a personal interview. Strength exercises included push-ups, sit-ups, dumbbells, weights and bars, and flexibility exercises included stretching and bare-handed gymnastics. PA was assessed by self-administered questionnaire with the question “On how many days did you spend more than 10 minutes engaged in intense physical activity in the last week?” and is expressed as the metabolic equivalent of task (MET)-hours per week (MET-h per week) according to the international PA questionnaire. In total, 3.3 METs were assigned for walking; 5.5 METs for moderate intensity activity, such as slow swimming, doubles tennis, volleyball, badminton, table tennis, and carrying light objects but not walking; and 8 METs for vigorous intensity activity, such as jogging, mountain climbing, fast biking, fast swimming, soccer, basketball, jumping rope, squash, singles tennis and carrying heavy items^60^.

### Statistical analysis

According to a previous study, loss of muscle mass and strength accelerates around age 50^23^, and stratified analyses to identify factors associated with sarcopenia in two age (<50, ≥50 years) groups were performed. The results are presented as the mean ± standard deviation (SD) or number (%). All analyses were carried out for male and female participants. To compare means and proportions between each group, Student’s t and χ2 tests were performed.

For the comparisons of factor variables and sarcopenia, logistic regression was performed after weighting all values without adjustment. Both univariate and multivariate logistic regression analysis models were used to examine the correlations of nutritional factors, EI/BMR, and PA with sarcopenia after adjusting for all possible confounding factors, such as age, disease (HTN, DM, and CCVD), lifestyle factors (smoking and alcohol consumption), and PA. The odds ratios (ORs) and 95% confidence intervals (CIs) were calculated.

To examine the nonlinear relationships of EI/BMR or MET-h/week with the risk of sarcopenia, we treated EI/BMR and MET-h/week as continuous variables in the regression models. The plotted average adjusted predictions of the risk of sarcopenia across the ranges of EI/BMR or PA (MET-h/week) were included in the regression model.

To determine the interactive effect of EI/BMR and PA on sarcopenia, we calculated postestimate standard errors and confidence intervals for the linear combinations of coefficients after logistic regression. The results are displayed as ORs. Two-sided p-values <0.05 were considered statistically significant. The entire analysis was performed using Stata ver. 15.1 (Stata Corp., College Station, TX, USA)

All experiments were performed in accordance with the relevant named guidelines and regulations.

## Supplementary information

Supplementary information.
