# Supplementary material for: Sex- and age-specific effects of energy intake and physical activity on sarcopenia
Source: Sci Rep. 2020 Jun 17;10:9822. doi: 10.1038/s41598-020-66249-6 (PMC7300112; doi:10.1038/s41598-020-66249-6)
Supplement: Supplementary file 1 — Supplementary information. [file 41598_2020_66249_MOESM1_ESM.docx]

Sex- and age-specific effect of energy intake

and physical activity on sarcopenia

Yu Jin Cho^a^, Youn-Hee Lim^b^, Jae Moon Yoon^a^, Hyung-Jin Yoon^c,d^, Minseon Park^a^*

a. Department of Family Medicine, Seoul National University, College of Medicine, Seoul, Korea

b. Institute of Environmental Medicine, Seoul National University Medical Research Center, Seoul, Korea

c. Department of Biomedical Engineering, Seoul National University College of Medicine, Seoul, Korea

d. Bio-MAX Institute, Seoul National University, Seoul, Korea

* Corresponding author: Minseon Park, Department of Family Medicine, Seoul National University, Hospital, Seoul National University College of Medicine, 101, Daehak-ro Jongno-gu, Seoul 03080, Korea

Tel: +82-2-2072-3497, Fax: +82-2-766-3276, E-mail: msp20476@hanmail.net

**Table S1.** Average proportion of energy intake, exercise and physical activity according to age and sex

|  | Age < 50 (N=8,092) | | | Age ≥ 50 (N=8,221) | | |
| --- | --- | --- | --- | --- | --- | --- |
| Characteristics | Male (N=3,127) | Female (N=4,965) | *P-value* | Male (N=3,449) | Female (N=4,772) | *P-value* |
| Intake:BMR ratio | 1.51±0.53 | 1.42±0.54 | <0.0001 | 1.50±0.51 | 15.50±0.53 | 0.890 |
|  |  |  |  |  |  |  |
| Strength exercise |  |  |  |  |  |  |
| Yes | 1,299 (41.67) | 922 (18.62) |  | 1,124 (32.75) | 622 (13.12) |  |
| No | 1,818 (58.33) | 4,030 (81.38) | <0.0001 | 2,308 (67.25) | 4,118 (86.88) | <0.0001 |
|  |  |  |  |  |  |  |
| Flexibility exercise |  |  |  |  |  |  |
| Yes | 1,787 (57.33) | 2,741 (55.35) | 0.081 | 1,661 (48.40) | 1,987 (41.92) | <0.0001 |
| No | 1,330 (42.67) | 2,211 (44.65) |  | 1,771 (81.60) | 2,753 (58.08) |  |
|  |  |  |  |  |  |  |
| MET-h/week | 58.06±86.84 | 42.16±68.27 | <0.0001 | 91.63±86.44 | 49.44±83.31 | <0.0001 |

**Figure S1.** Predicted physical activity levels according to energy intake

**
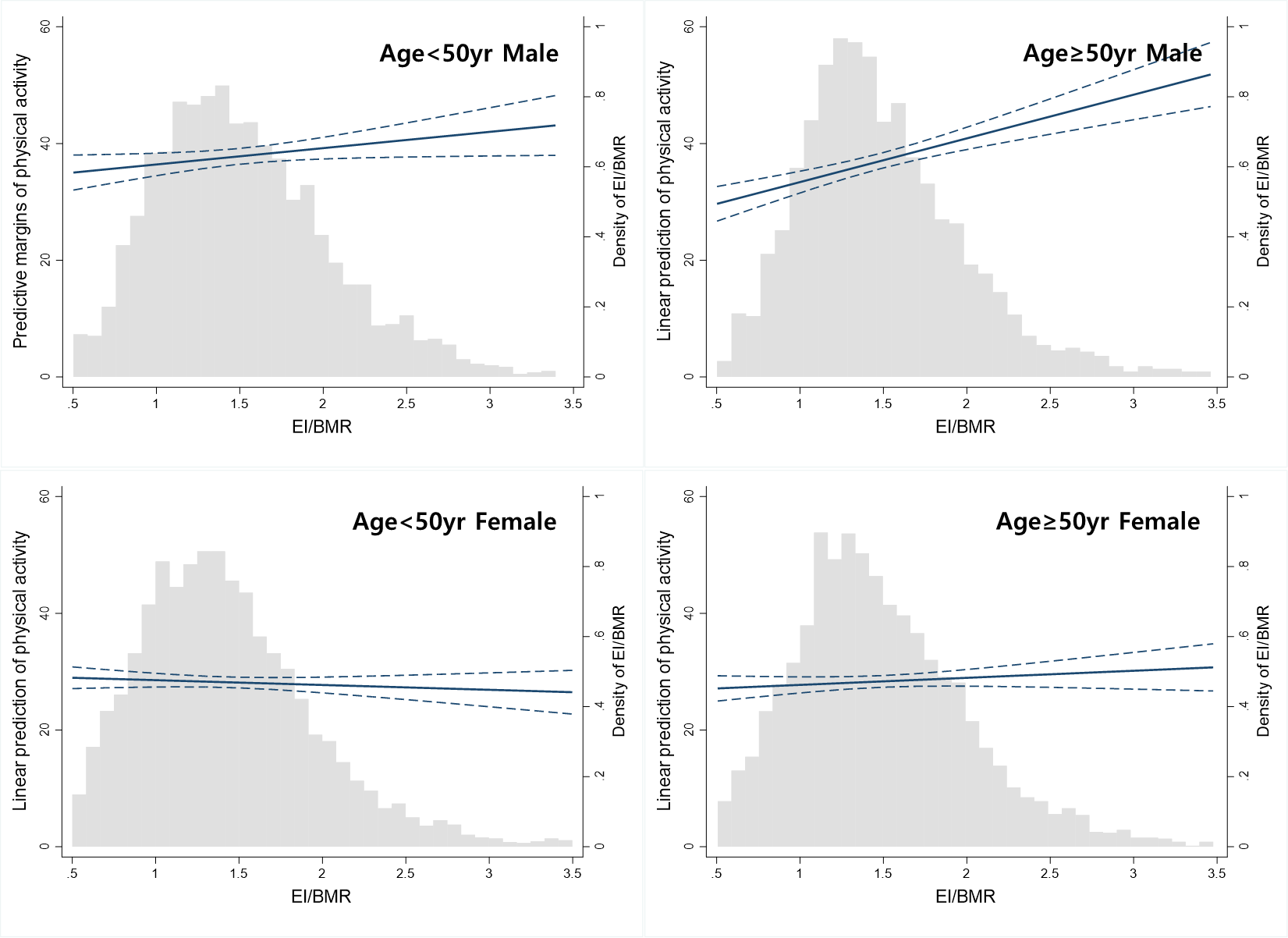
**

**Figure S2.** Predicted physical activity levels according to energy intake **
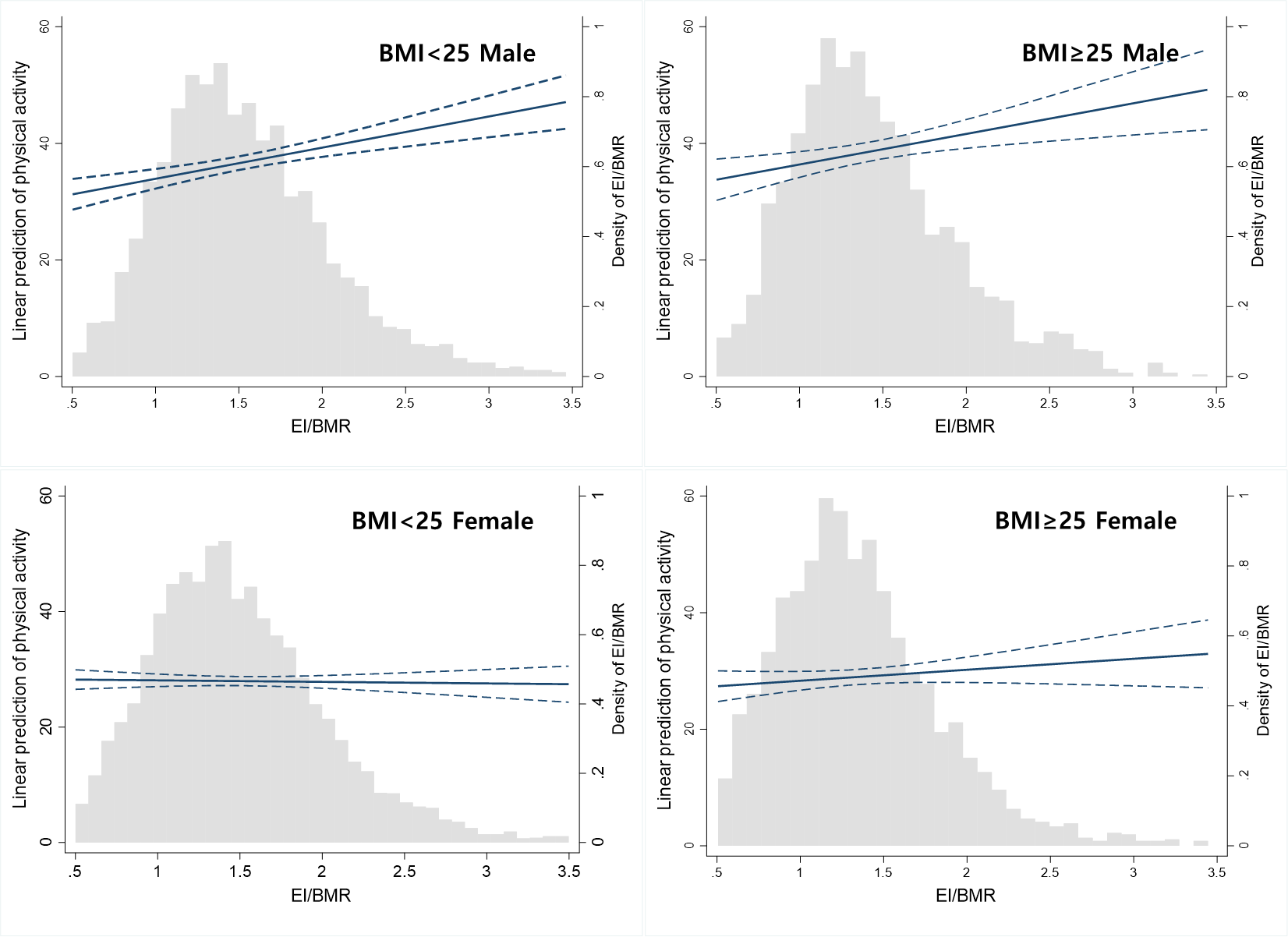
**
